# Increased Skeletal Muscle Fiber Cross-Sectional Area, Muscle Phenotype Shift, and Altered Insulin Signaling in Rat Hindlimb Muscles in a Prenatally Androgenized Rat Model for Polycystic Ovary Syndrome

**DOI:** 10.3390/ijms21217918

**Published:** 2020-10-25

**Authors:** Auryana DeChick, Rebecca Hetz, Jack Lee, Diana L. Speelman

**Affiliations:** Department of Biochemistry, Lake Erie College of Osteopathic Medicine, Erie, PA 16509, USA; adechick53195@med.lecom.edu (A.D.); rhetz@lecom.edu (R.H.); jlee@lecom.edu (J.L.)

**Keywords:** hyperandrogenemia, muscle fiber type, muscle cross-sectional area, intra-muscular lipids, peri-muscular lipids, PCOS, insulin signaling, insulin resistance, IRS1

## Abstract

Women with polycystic ovary syndrome (PCOS) are reported to have greater lean mass and insulin resistance. To examine muscular changes in a prenatally androgenized (PNA) rat model for PCOS, Sprague–Dawley rats were exposed to 5 mg testosterone or vehicle daily on gestational days 16–19. At 15 weeks of age, endurance on a rota-rod treadmill was measured. At 16 weeks of age, fasting blood glucose and insulin, hindlimb skeletal muscle mass, muscle fiber cross-sectional area (CSA) and composition, and intra- and peri-muscular lipid droplets were examined. Expression of mitochondrial marker ATP synthase and insulin signaling proteins were also investigated. Compared with controls, PNA female rats demonstrated greater total body and hindlimb muscle weights, greater muscle fiber CSA, and trending reduced time on the rota-rod. An increase in fibers co-expressing the slow and fast isoforms of myosin (90 vs. 86%, *p* < 0.05) and greater expression of ATP synthase (6-fold, *p* < 0.005) were observed in the gastrocnemius (GN) muscle. More lipid content was observed in GN and tibialis anterior (TA) muscles. PNA rats had elevated fasting serum insulin (1.9 vs. 1.2 ng/mL, *p* < 0.005) but comparable fasting glucose. Expression of total and Ser^636/9^-phosphorylated IRS1 were altered in PNA rat hindlimb muscles. Together, skeletal muscle alterations in hindlimb muscles of a PNA rat model for PCOS may represent consequences of, or adaptations to, insulin resistance in this model.

## 1. Introduction

Polycystic ovary syndrome (PCOS) is the most common endocrinopathy in women, affecting female reproductive, metabolic, and psychological health from puberty to menopause and beyond [1,2,3]. Evidence supports a key role for elevated levels of androgens in the pathogenesis of PCOS [4,5,6,7,8,9,10,11], which can influence the function of various tissues in the body, including skeletal muscle. Androgen levels are associated with muscle size and strength [12,13,14]. Women with PCOS are reported to have increased lean mass [15,16,17], greater muscle strength [18,19], and enhanced muscle strength after progressive resistance training [20], suggesting a relationship between elevated androgen levels and muscle structure or function in these women. Muscle size is reported to positively correlate with serum androgen levels in women with PCOS [18,21,22], although some did not find a relationship between lean mass and androgen levels [15,16]. Some reports indicate that insulin resistance—which is common in women with PCOS [23,24,25,26]—rather than high androgen levels, correlates with increased lean mass in this population [15,16,27]. Studies reporting a positive correlation between insulin resistance and increased lean mass in women with PCOS did not explore the composition of skeletal muscle in participants. Skeletal muscle fiber composition can influence force generated, exercise capacity, and fuel usage. In addition, it may influence whole-body glucose tolerance and serum insulin levels [28,29].

Prenatally androgenized (PNA) rodent models exhibit many of the characteristics associated with PCOS in humans, including disrupted reproductive cycles, hyperandrogenemia, polycystic ovarian morphology, and metabolic dysfunction [11,30,31,32,33]. PNA model androgen exposure during this prenatal period mimics the testosterone surge that is observed in male rats [34] and results in elevated testosterone and androstenedione in adult female rats [33]. Such models are conducive both to biochemical and structural analysis of multiple tissues, as well as pharmacologic and nonpharmacologic manipulation in a controlled environment. Skeletal muscle structure and function has not been fully characterized in these rodent models for PCOS but is hypothesized to mimic the increased mass and insulin resistance reported in women with the disorder. The aim of this study was to test the hypothesis that the skeletal muscle of the PNA rodent model mimics the increased mass and insulin resistance in women with the PCOS. We examined hindlimb muscle mass and cross-sectional area and muscle composition in a prenatally androgenized rat model for PCOS. In addition, we examined fasting blood glucose and serum insulin levels and expression of insulin signaling proteins in hindlimb skeletal muscles.

## 2. Results

### 2.1. Body Weight

**Figure 1:**
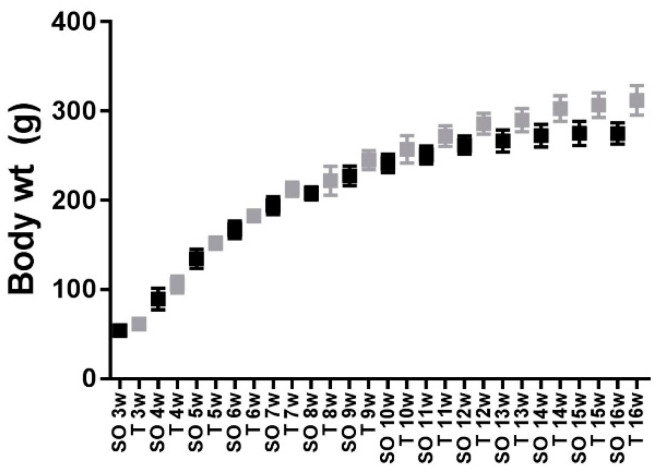
Prenatally androgenized female rats weighed more than control rats beginning at 4 weeks of age. Sprague–Dawley rats were prenatally exposed to sesame oil (*n* = 15) or 5 mg testosterone in sesame oil (*n* = 9) each day from gestation days 16–19. Rats were weighed weekly from 3 weeks to 16 weeks of age. Beginning at 4 weeks of age, the prenatally androgenized (PNA) rats (gray squares) weighed significantly more than control rats (black squares). Values are means ± SD. One-way ANOVA with Sidak’s multiple comparisons test, *p* < 0.0001.

Beginning at 4 weeks and continuing through 16 weeks of age, female rats prenatally exposed to testosterone weighed significantly more than control rats (Figure 1, SO vs. T, *p* < 0.0001). This difference became more pronounced at 14 through 16 weeks of age.

### 2.2. Rota-Rod Time Trials

**Figure 2:**
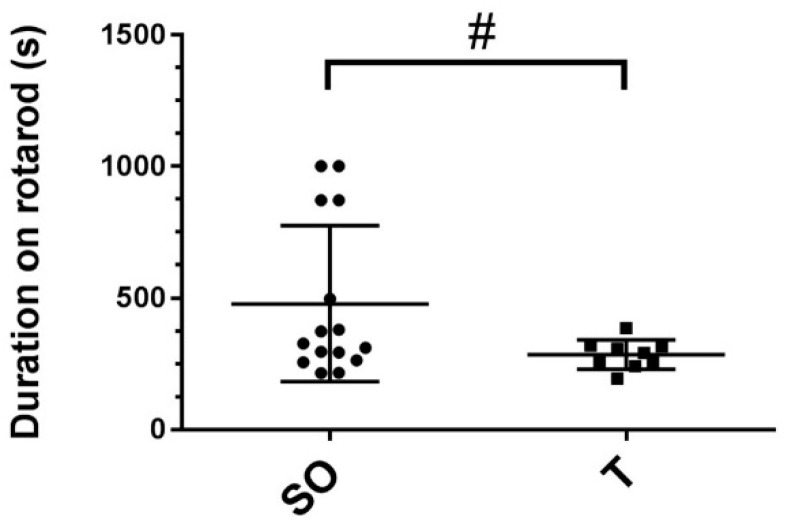
Rota-rod exercise times trended toward shorter duration in PNA compared with control rats. At 15 weeks of age, female rats that were prenatally exposed to sesame oil (SO, *n* = 15) or testosterone (T, *n* = 9) were placed on a rota-rod that gradually accelerated to a maximum speed of 40 rpm. The time spent on the rota-rod before falling off was recorded, with a maximum possible time of 1000 s. Each rat underwent a timed trial once daily for three consecutive days. Data were analyzed using unpaired t-tests and trended toward shorter duration for PNA rats (# *p* = 0.0684). Values are the means ± SD.

To determine if control and PNA rats exhibited a difference in exercise endurance, rats were placed on an accelerating rota-rod treadmill. The time spent walking on the rota-rod before falling off, up to a maximum of 1000 s, was recorded for each rat for three consecutive days. As shown in Figure 2, compared to control rats, female rats prenatally exposed to testosterone exhibited a trend toward less time on the rota-rod (438 vs. 236 s, *p* = 0.07). However, whereas all testosterone-exposed rats stayed on the rota-rod treadmill for 195–390 s, some rats in the control group stayed on for a similar amount of time (215–380 s), while others stayed on for over 870 s (Figure 2).

### 2.3. Hindlimb Muscle Weights

**Figure 3:**
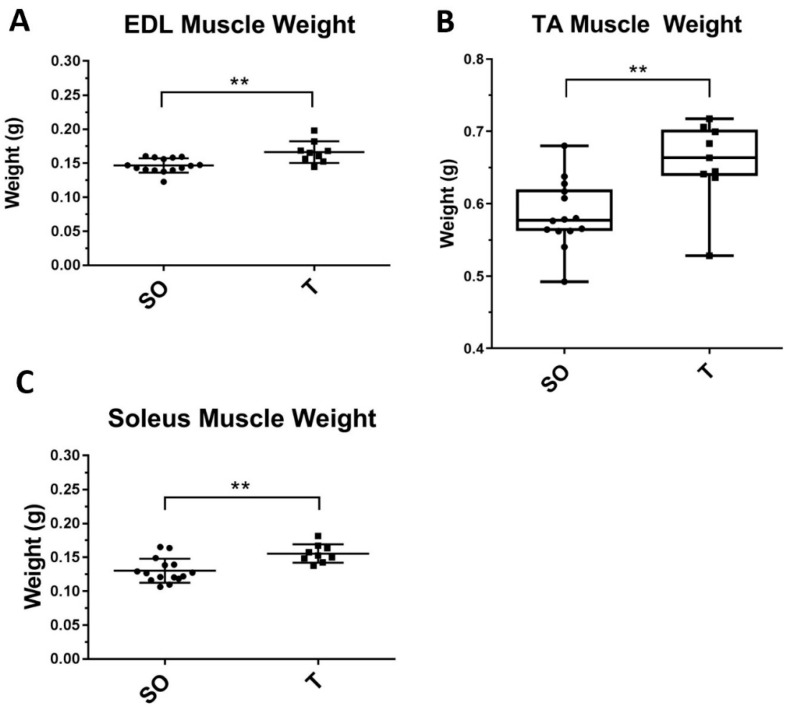
Greater hindlimb skeletal muscle mass in PNA rats compared to control rats. At 16 weeks of age, animals were euthanized and hindlimb muscles were carefully dissected from female rats prenatally exposed to sesame oil (SO, *n* = 15) or testosterone (T, *n* = 9) prior to weighing. Data with normal distribution were analyzed by unpaired t-tests (**A**,**B**), and those not normally distributed were analyzed by Mann–Whitney U test (**C**). Values are means ± SD (**A**,**B**) or quartiles with the median shown (**C**). ** *p* < 0.005.

In addition to greater body weight, PNA rats also exhibited greater tibialis anterior (TA), extensor digitorum longus (EDL), and soleus (SOL) muscle weights than control rats at 16 weeks of age (Figure 3, *p* < 0.005).

### 2.4. Hindlimb Muscle Fiber Cross-Sectional Area

**Figure 4:**
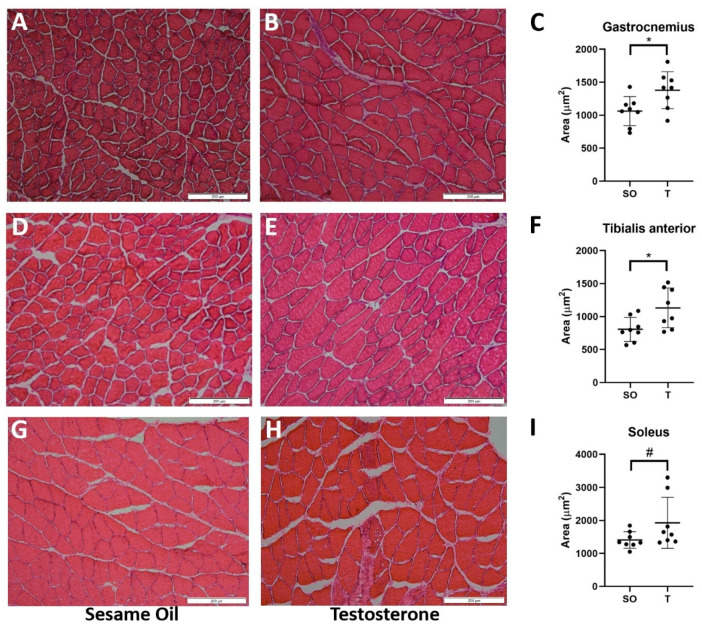
Greater hindlimb skeletal muscle cross-sectional area in PNA rats. At 16 weeks of age, animals were euthanized, and hindlimb muscles were carefully dissected. Gastrocnemius (GN), tibialis anterior (TA), and soleus (SOL) muscles from female rats exposed to sesame oil (*n* = 8) or testosterone (*n* = 8) were snap-frozen and cryosectioned at 10 µm thickness prior to fixing in paraformaldehyde and staining with hematoxylin and eosin. For each rat, 3 distinct fields per muscle sample were imaged at 200x magnification; representative images from muscles from rats prenatally exposed to sesame oil (**A**,**D**,**G**) or testosterone (**B**,**E**,**H**) are shown. Fiber cross-sectional area was measured; data were normally distributed and analyzed using unpaired t-tests (**C**,**F**,**I**). Values represent the mean ± SD. * *p* < 0.05, # *p* = 0.0924. Scale bars, 200 µm.

To determine if the greater mass of hindlimb muscles correlated with increased fiber size, we next examined cross-sectional area (CSA) for several hindlimb muscles. As shown in Figure 4, compared with control rats, the testosterone-exposed rats exhibited greater CSA in the GN (1062 vs. 1379 µm^2^, *p* = 0.02), TA (807 vs. 1131 µm^2^, *p* = 0.02), and SOL (1409 vs. 1927 µm^2^, *p* = 0.09) muscles.

### 2.5. Hindlimb Muscle Fiber Composition

**Figure 5:**
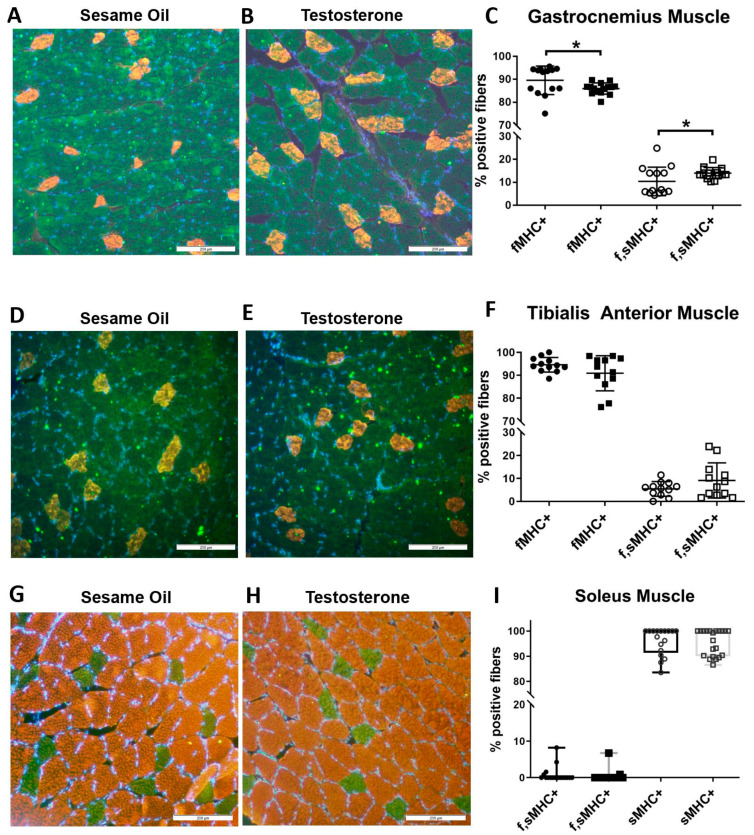
More skeletal muscle fibers in PNA rat gastrocnemius muscle are positive for both the fast and slow isoforms of myosin heavy chain. Gastrocnemius (**A**–**C**), tibialis anterior (**D**–**F**), and soleus (**G**–**I**) skeletal muscles from 16-week-old female rats exposed to sesame oil (*n* = 4–5) or testosterone (*n* = 4–5) were cryosectioned at 20 µm thickness prior to indirect immunolabeling for the fast (green) and slow (red) isoforms of myosin heavy chain. Blue indicates DAPI-stained nuclei. For each rat, 3 distinct fields per muscle sample were imaged at 200× magnification and used to determine the percent of fibers that expressed the slow myosin heavy chain isoform (sMHC+, red), fibers that expressed the fast myosin heavy chain isoform (fMHC+, green), or fibers that expressed both fast and slow isoforms (f,sMHC+, green, and red). Scale bars, 200 µm. Data were analyzed using unpaired t tests (GN and TA) or Mann–Whitney U tests (SOL). (**A**–**C**) In gastrocnemius muscles, the predominant fiber type was fMHC+. Compared to the controls (closed and open circles), PNA rats (closed and open squares) had a small but significant increase in fibers expressing both the fast and slow isoforms of MHC (f,sMHC+), with a concomitant decrease in fibers expressing only fMHC. TA muscles had a similar fiber composition as the GN muscles, but no significant difference in fiber composition between control and PNA rats. No significance or trend was observed for soleus muscles (**G**–**I**; SO, black boxes and T, gray boxes), which consisted primarily of sMHC+ fibers. Values are means ± standard deviation (**C**,**F**) or quartiles with the median shown (I). * *p* < 0.05.

Next, we examined muscle fiber type composition of GN and TA (consisting of a mixture of type I and type II fibers) and SOL (consisting of predominantly type I fibers) muscles by immunolabeling for the fast and slow isoforms of myosin heavy chain (fMHC and sMHC, respectively). PNA rats exhibited significantly more fibers expressing both isoforms (f,sMHC+, 10% vs. 14%) and fewer expressing only the fast isoform (fMHC+, 90% vs. 86%) in the GN muscle (Figure 5A–C, *p* < 0.05). Type I fibers (sMHC+) in this muscle represented less than 1% of all fibers.

There was no significant change in fiber composition of TA muscles, although some PNA rats did exhibit more fibers expressing both isoforms and fewer expressing only the fast isoform (Figure 5D–F; *p* = 0.14). No significant difference was seen in the SOL muscles, which are predominantly composed of type I fibers (Figure 5G–I). 

### 2.6. Mitochondrial Marker ATP Synthase Expression in Hindlimb Muscle

**Figure 6:**
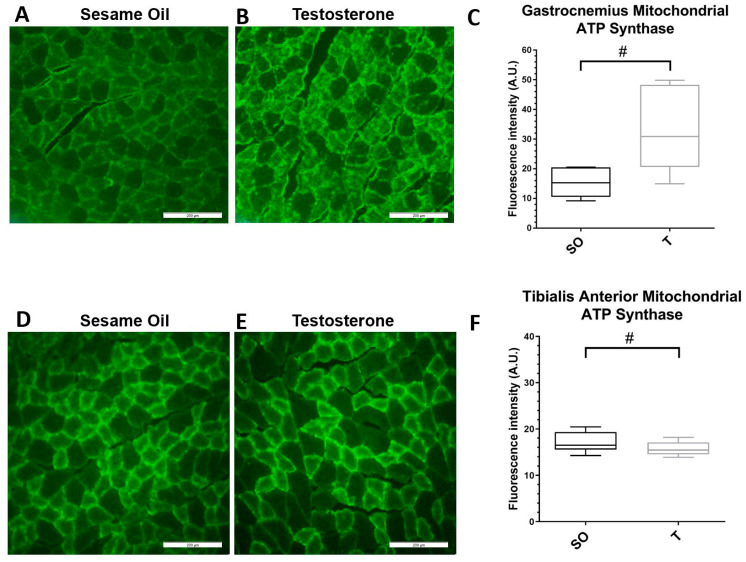

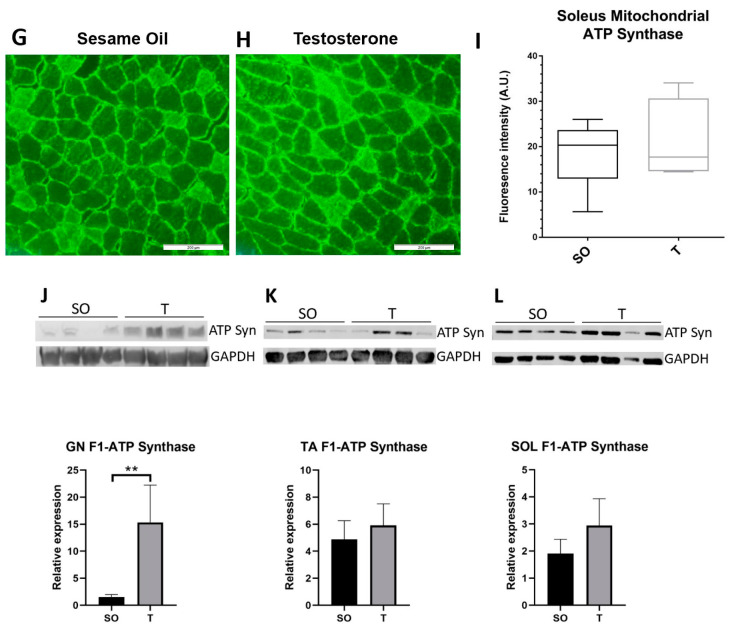
Expression of the mitochondrial marker ATP synthase is higher in gastrocnemius muscle in PNA rats. Gastrocnemius (**A**–**C**), tibialis anterior (**D**–**F**), and soleus (**G**–**I**) skeletal muscles from 16-week-old female rats exposed to sesame oil (*n* = 4–5) or testosterone (*n* = 4–5) were cryosectioned at 20 µm thickness prior to indirect immunolabeling for ATP synthase. Scale bars, 200 µm. For each rat, 3 distinct fields per muscle sample were imaged and the fluorescence intensity quantified (**C**,**F**,**I**). Fluorescence intensity data were analyzed using a Mann–Whitney U test. Fluorescence intensity trended higher in muscles from PNA rats for gastrocnemius (# *p* = 0.0556), trended slightly lower in TA (# *p* = 0.0979), but was not significantly different in soleus muscle. Quartiles with the median are shown. (**J**–**L**) Protein expression was examined by Western blot and quantified by densitometric analysis. Samples of (**J**) GN, (**K**) TA, and (**L**) SOL muscles from rats exposed to sesame oil (*n* = 8) or testosterone (*n* = 8) were separated by SDS-PAGE and transferred to nitrocellulose prior to detection with antibodies to the alpha subunit of ATP synthase or GAPDH (top panels). Values for ATP synthase were normalized to GAPDH and analyzed using an unpaired t test or Mann–Whitney U test, which indicated significantly greater expression of ATP synthase in GN, but not TA or SOL, from PNA rats (bottom panels). Values shown are means with SD. ** *p* < 0.005.

We next examined the expression of ATP synthase, a mitochondrial protein, in GN, TA, and SOL muscles. There was a trend towards greater ATP synthase immunofluorescence in the GN muscle (Figure 6A–C, *p* = 0.06) and a slight decrease in TA muscle (Figure 6D–F, *p* = 0.09). There was no difference in fluorescence intensity in the SOL muscles between the control and PNA rats (Figure 6G–I). Further examination of ATP synthase protein expression by Western blot revealed an approximately 6-fold increase in ATP synthase expression in GN muscles from PNA rats (Figure 6J, *p* = 0.004). However, there was no significant increase in ATP synthase protein expression in either TA or SOL muscle by Western blot analysis (Figure 6K,L).

### 2.7. Lipid Content in Hindlimb Muscle

**Figure 7:**
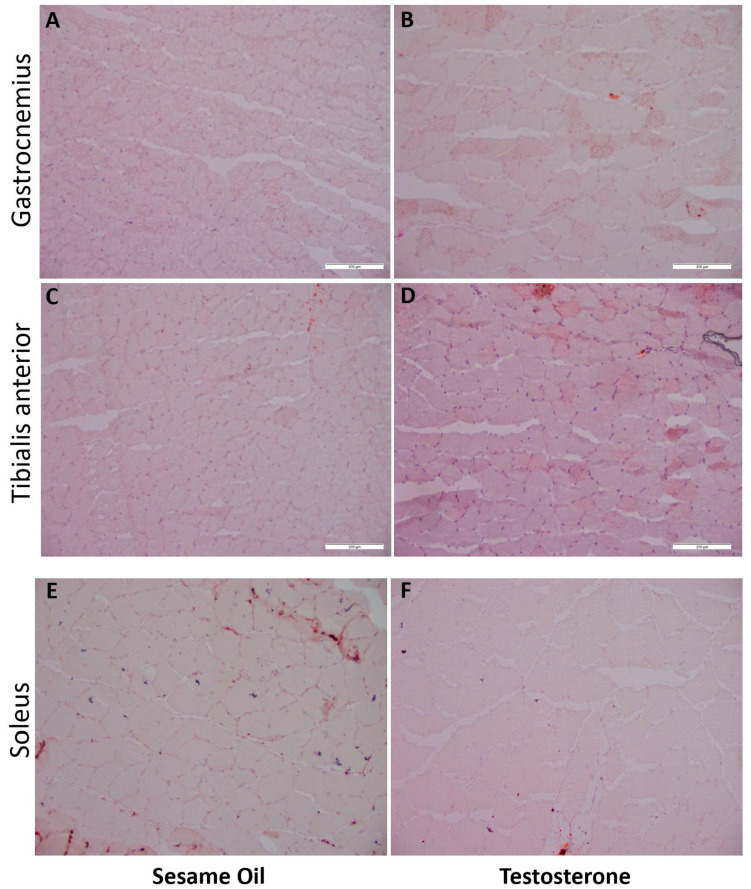
More Oil Red O staining of lipids in gastrocnemius and tibialis anterior muscles from PNA rats. Gastrocnemius, tibialis anterior, and soleus muscles from 16-week-old rats prenatally exposed to sesame oil (*n* = 8) or testosterone (*n* = 8) were snap-frozen and cryosectioned at 10 µm thickness prior to fixing in paraformaldehyde and staining for lipids with Oil Red O (ORO). Scale bars, 200 µm. For each rat, 2–4 distinct fields per muscle sample were imaged at 200× magnification; representative images from muscles from rats prenatally exposed to sesame oil (**A**,**C**,**E**) or testosterone (**B**,**D**,**F**) are shown. More prominent intra-muscular and peri-muscular staining with ORO was noted in the GN and TA muscles from PNA rats. No difference was appreciated between control and PNA rats in soleus muscles.

In order to examine intra-muscular and peri-muscular lipid content, which is associated with insulin resistance [35], we used Oil Red O (ORO) to stain lipid content in muscle sections from GN, TA, and SOL muscles. Compared with controls, there was more intra-muscular and peri-muscular ORO staining in PNA rat GN and TA muscles (Figure 7). No differences were appreciated in SOL muscle.

### 2.8. Fasting Blood Glucose and Serum Insulin

**Figure 8:**
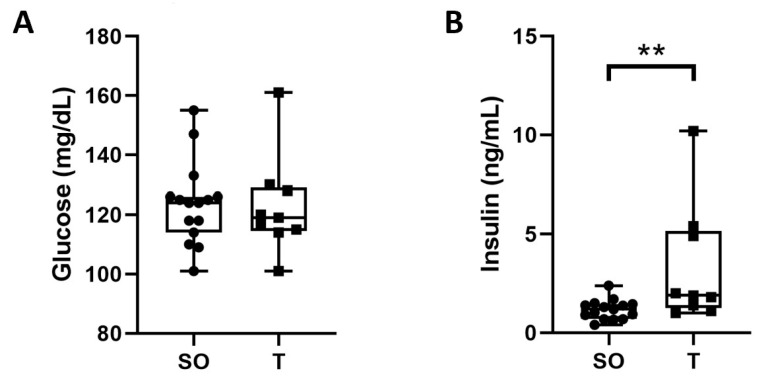
Elevated fasting serum insulin in adult PNA rats. Tail vein blood samples were taken from control (*n* = 15) and PNA (*n* = 9) rats at 16 weeks of age following an overnight fast. (**A**) A drop of blood was used to determine blood glucose using a standard glucometer. (**B**) Serum insulin concentrations were measured by ELISA. While fasting blood glucose levels were comparable, fasting serum insulin was significantly higher in the PNA rats. Data were analyzed by Mann–Whitney U tests and are shown as quartiles with the median. ** *p* < 0.005.

We next sought to determine if there might be changes in fasting blood glucose and serum insulin in the PNA rats compared to the control rats, in addition to the observed differences in muscle CSA, fiber type, and intra- and peri-muscular lipid content. Fasting blood glucose levels were not significantly different between the control and PNA rats at 16 weeks of age (Figure 8A). However, fasting serum insulin was significantly higher in PNA rats (Figure 8B, 1.2 vs. 1.9 ng/mL, *p* = 0.008).

### 2.9. Expression of Insulin Signaling Proteins in Hindlimb Muscle

Skeletal muscle plays a major role in insulin-mediated glucose uptake. Given the higher levels of fasting serum insulin in adult rats, we next examined the expression of insulin signaling pathway proteins in the GN, TA, and SOL muscles. 

**Figure 9:**
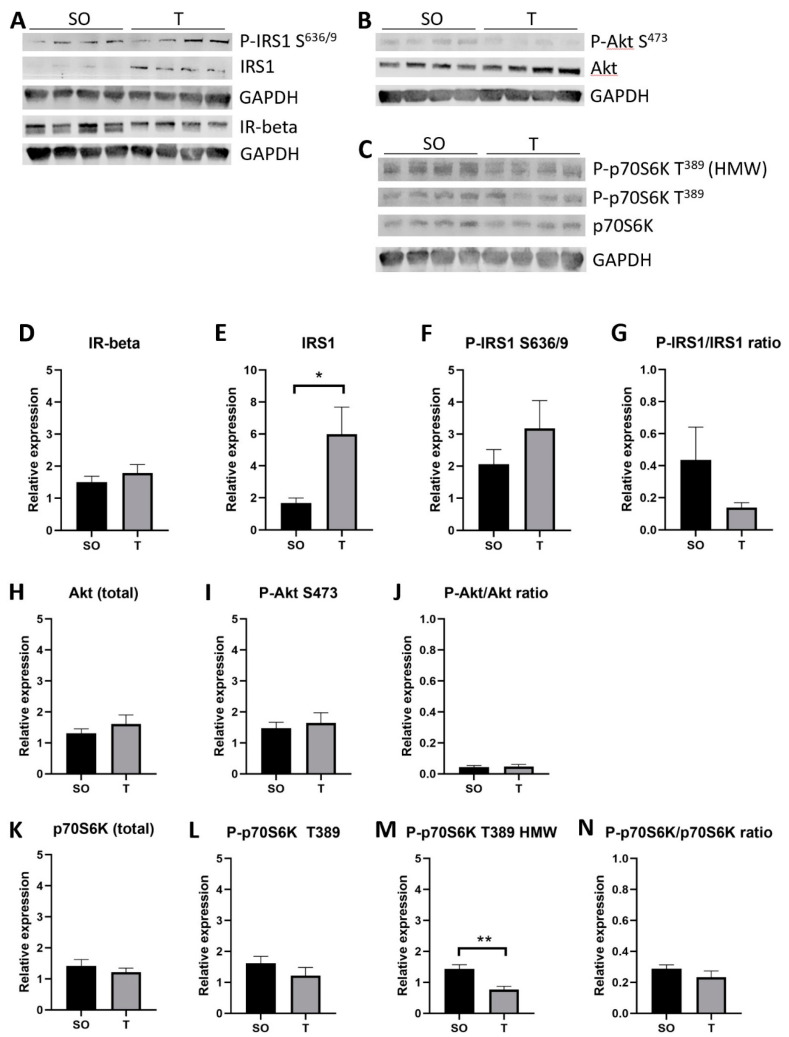
Greater IRS1 protein expression in PNA gastrocnemius muscle. Gastrocnemius muscles from 16-week-old rats were homogenized on ice in buffer containing protease and phosphatase inhibitors. (**A**–**C**) Homogenates (30 µg) were separated by SDS-PAGE prior to transfer to nitrocellulose for immunoblot analysis of proteins. (**D**–**N**) Band intensity was quantified using ImageStudio Lite software (Version 5.2), and data were analyzed using unpaired t tests. Expression of total IRS1 protein was greater in GN from PNA rats (**A**,**E**). There appeared to be a decrease in the ratio of Ser^636/9^ phosphorylated IRS1 to total IRS1 (**G**), though this was not statistically significant. GN from PNA rats expressed less T^389^ phosphorylated p70S6K protein (**C**,**M**). Means with SD are shown. * *p* < 0.05, ** *p* < 0.005.

In GN (Figure 9), there was an approximately 3.6-fold increase in the expression of IRS1 in PNA rats (*p* = 0.03), and the ratio of P-IRS1 S^636/9^ to IRS1 appeared to decrease by nearly half (*p* = 0.16). In addition, the expression of a high molecular weight (HMW) Thr^389^ phosphorylated p70S6K was nearly 50% lower in PNA rats (*p* = 0.002), although the total p70S6K and ratio of P-p70S6K T^389^ to p70S6K did not change. No significant changes were detected in IR-beta, P-IRS1 S^636/9^, P-Akt S^473^, Akt, or the ratio of P-Akt S^473^ to Akt in GN muscle.

**Figure 10:**
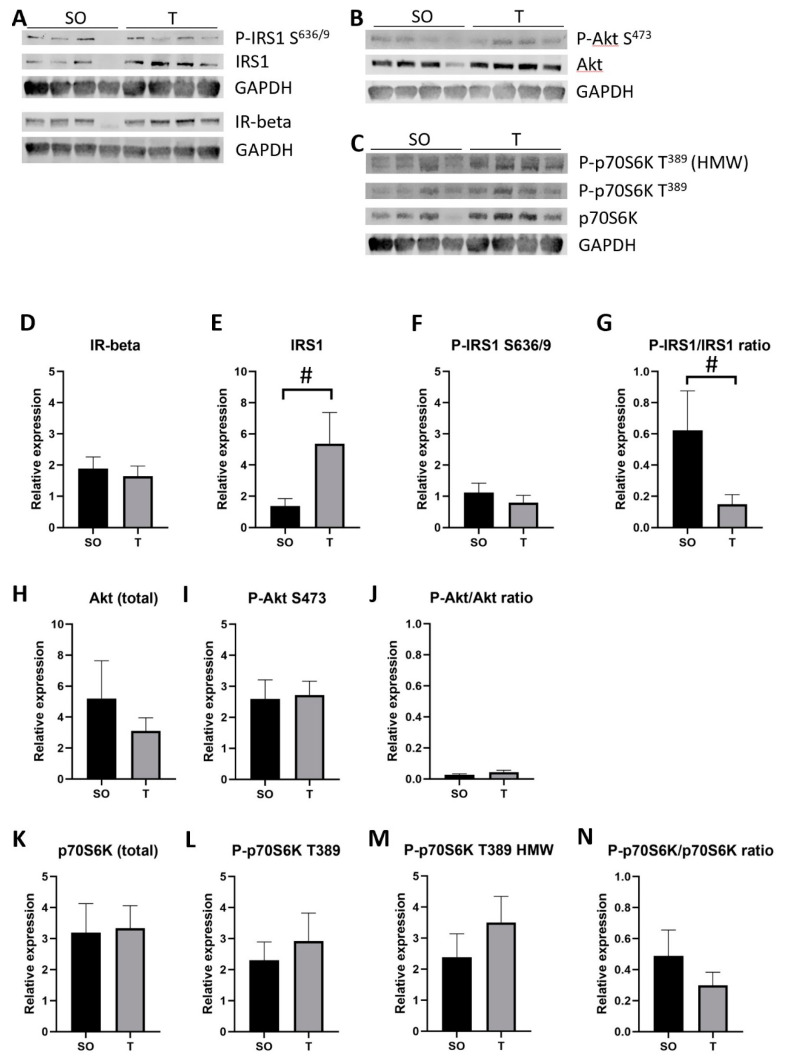
Greater IRS1 protein expression and lower Ser^636/9^ phosphorylated IRS1 to IRS1 ratio in PNA tibialis anterior muscle. Tibialis anterior muscles from 16-week-old rats were homogenized on ice in buffer containing protease and phosphatase inhibitors. (**A**–**C**) Homogenates (30 µg) were separated by SDS-PAGE prior to transfer to nitrocellulose for immunoblot analysis of proteins. (**D**–**N**) Band intensity was quantified using ImageStudio Lite software (Version 5.2), and data were analyzed using unpaired t tests. Expression of total IRS1 protein trended higher in TA from PNA rats (**A**,**E**; # *p* = 0.0911); Ser^636/9^ phosphorylated IRS1 expression was comparable, resulting in a trend toward decreased P-IRS1 to total IRS1 ratio in TA from PNA rats (**G**; # *p* = 0.0754). Means with SD are shown.

In TA (Figure 10), there was a trend toward a 3.9-fold increase in IRS1 in PNA rats (*p* = 0.09), with a trend toward a 75% reduction in the ratio of P-IRS1 S^636/9^ to IRS1 (*p* = 0.08). No significant difference was detected in IR-beta, P-IRS1 S^636/9^, P-Akt S^473^, Akt, the ratio of P-Akt S^473^ to Akt, P-p70S6K T^389^, total p70S6K, or the ratio of P-p70S6K T^389^ to p70S6K.

**Figure 11:**
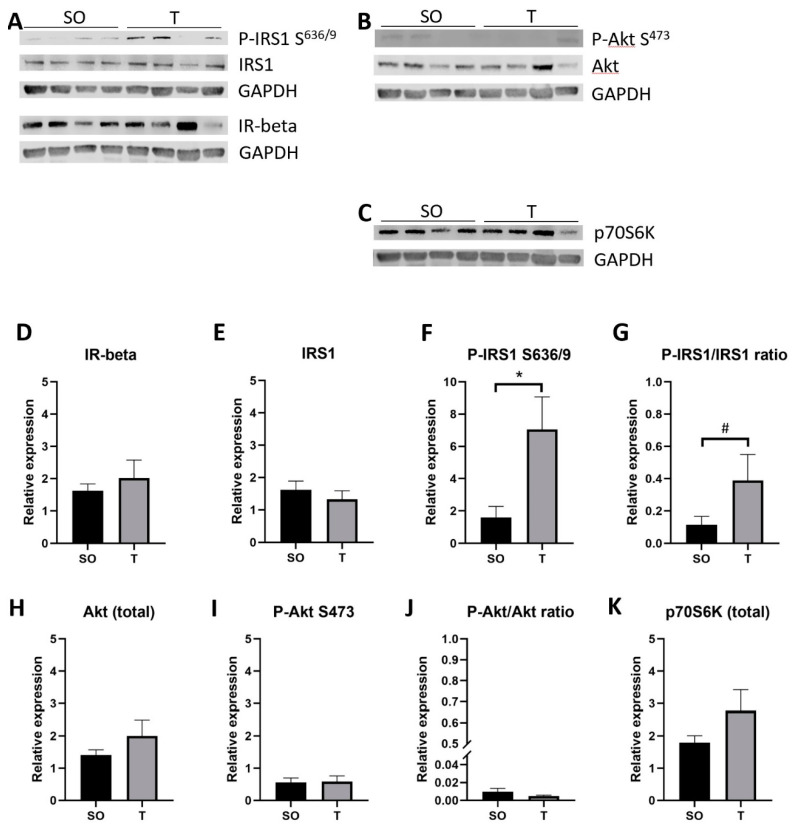
Greater Ser^636/9^ phosphorylated IRS1 expression in PNA soleus muscle. Soleus muscles from 16-week-old rats were homogenized on ice in buffer containing protease and phosphatase inhibitors. (**A**–**C**) Homogenates (30 µg) were separated by SDS-PAGE prior to transfer to nitrocellulose for immunoblot analysis of proteins. (**D**–**K**) Band intensity was quantified using ImageStudio Lite software (Version 5.2), and data were analyzed using unpaired t tests. Expression of Ser^636/9^ phosphorylated IRS1 protein was greater in soleus from PNA rats (**A**,**E**); IRS1 expression was comparable, resulting in a trend toward increased P-IRS1 to total IRS1 ratio in soleus from PNA rats (**G**; # *p* = 0.10). Means with SD are shown. * *p* < 0.05.

In SOL (Figure 11), there was a 4.4-fold increase in P-IRS1 S^636/9^ (*p* = 0.02) and a trend toward a 3.9-fold higher P-IRS1 S^636/9^ to IRS1 ratio (*p* = 0.10) in PNA rats. No significant difference was detected in IR-beta, IRS1, P-Akt S473, Akt, the ratio of P-Akt S^473^ to Akt, or total p70S6K. P-p70S6K T^389^ was unable to be detected in SOL samples.

## 3. Discussion

Prenatally androgenized rodent models have demonstrated the reproductive and metabolic features associated with PCOS [11,30,31,32,33]. To our knowledge, this is the first study to demonstrate altered hindlimb skeletal muscle fiber cross-sectional area and composition, in conjunction with changes in insulin signaling pathway protein expression, in a PNA rat model for PCOS. These findings lend further support to the use of this model to study PCOS and suggest that exposure to excess androgens in females may alter skeletal muscle structure and function. As skeletal muscle accounts for a large proportion of body mass and metabolic activity, changes in muscle structure and function may influence fuel handling and contribute to altered metabolic function. 

### 3.1. Body Composition, Muscle Structure, and Function in PCOS

Androgens are well-known to increase muscle mass and strength [12,13,14]. Women with PCOS demonstrate hyperandrogenemia [36,37,38], which may influence muscle structure and function in this population. Analyses of body composition in women with PCOS compared to those without have suggested increased lean mass [15,16,17] and greater leg muscle mass in women with PCOS [39]. Some have reported that the increased muscle mass correlates with androgen levels [21,39], while others did not find androgens to be a major correlate of increased lean mass [15,16]. Regarding muscle strength, while some did not find a significant difference [40], others have reported that women with PCOS have increased baseline muscle strength in multiple muscle groups compared to women without the disorder, irrespective of body composition [18]. Furthermore, a 4-month progressive resistance training regimen resulted in a greater increase in maximum strength in women with PCOS compared to controls, despite similar improvements in body composition (decreased body fat and increased lean body mass) and reductions in androgen levels in both groups [20].

In our PNA rat model for PCOS, female rats prenatally exposed to testosterone demonstrated significantly higher levels of testosterone and androstenedione at 16 weeks of age compared to controls [33]. Elevated levels of androgens may have resulted in greater hindlimb muscle mass and fiber cross-sectional area in our animals, suggestive of muscle hypertrophy. We found that PNA female rats exhibited greater overall body weight that became more pronounced as the animals aged, although we did not analyze body composition. Therefore, the increased body weight may reflect increased muscle mass as well as increased mass of adipose depots or other organs. Future directions should include examining skeletal muscle force transduction and fatigability in this PNA rat model for PCOS, in addition to body composition analysis such as by DEXA scan. 

### 3.2. Exercise in Humans with, and Animal Models for, PCOS

We examined exercise duration on a rota-rod treadmill and found that while some control rats stayed on the treadmill for a shorter (<400 s) and others longer (>800 s) period of time, PNA rats were only on the treadmill for a shorter duration (<400 s). This difference may indicate a reduced capacity for exercise in these animals or reduced motivation for staying on the treadmill. In a study using mice, PNA mice given free access to a running wheel were found to have reduced voluntary exercise activity compared to controls. Interestingly, this effect was eliminated by ovariectomy, suggesting that reduced voluntary exercise in this model may be partly mediated by ovarian hormones [41]. In humans, adolescent females with PCOS were found to engage less in physical activities than those without PCOS, and to exhibit less frequency and intensity when they do participate [42]. Together, data from human studies and animal models suggest that androgen excess in females may reduce voluntary exercise, suggesting that there may be some biological differences that influence exercise and which could explain our finding that all PNA rats spent less time on the rota-rod treadmill. 

With respect to exercise capacity, cross-sectional studies of overweight and obese women found that women with PCOS had similar VO_2_max compared with age- and BMI-matched controls [40,43]. However, a study of young (21 ± 2 years) women with PCOS found significant reductions in VO_2_max, oxygen consumption at aerobic threshold, and maximal workload compared to age- and BMI-matched controls, suggesting that some women with PCOS may have reduced exercise capacity [44]. Although we did not measure physiologic parameters of exercise capacity in the present study, the slight shift toward faster oxidative glycolytic (f,sMHC+) fibers and increased mitochondrial ATP synthase suggests that decreased exercise capacity in the PNA rats is unlikely. 

### 3.3. Muscle Fiber Composition and Metabolic Disorders

In a study with human subjects with metabolic syndrome, a shift in muscle composition was found in muscle biopsies taken from the vastus lateralis, including greater percentage of type IIa fast oxidative glycolytic muscle fibers and increased expression of ATP synthase in those fibers. In addition, there was a reduced percentage of type I slow oxidative fibers that correlated with greater insulin resistance [29]. Similarly, previous studies have shown that individuals with type 2 diabetes have a reduced percentage of type I fibers [45,46]. These findings are consistent with our own data that indicate a small but significant shift towards faster oxidative glycolytic (f,sMHC+) fibers and increased ATP synthase expression in the gastrocnemius muscle in PNA rats. These changes in hindlimb muscles may represent an adaptive phenotypic shift towards greater oxidative potential. The reported relationship between reduced type I fibers and insulin resistance suggest that, despite a possible compensatory mechanism to upregulate oxidation in type IIa fibers in the PNA rat, this shift may not be sufficient to prevent the development of metabolic dysfunction that has been reported in this model [32]. The mechanisms involved in and the physiological significance of this fiber-type shift warrants further investigation. Given the prevalence of insulin resistance and metabolic dysfunction in women with PCOS [47], future studies should determine if women with PCOS exhibit a similar alteration in muscle fiber composition. 

In addition to a slight but significant shift in muscle fiber composition of some hindlimb skeletal muscles toward a fast oxidative glycolytic phenotype, we also observed increased presence of lipid content in these muscles. Peri-muscular adipose increases with aging and obesity [48], and expansion of peri-muscular and intermuscular adipose correlates with skeletal muscle macrophage and T cell infiltration and insulin resistance in human subjects with obesity and in mice fed a high-fat diet [49]. In healthy, early postmenopausal women, thigh intermuscular adipose inversely correlated with insulin sensitivity index (ISI), whereas thigh subcutaneous adipose tissue positively correlated with ISI, independent of visceral adipose tissue [50]. In patients with obesity or type 2 diabetes, intra- and intermuscular adipose strongly correlated with insulin resistance, despite accounting for a relatively small percentage of the adipose mass in the thigh [51] or paraspinal muscles [52]. In addition, individuals with higher levels of insulin resistance demonstrated altered postprandial skeletal muscle handling and greater levels of intramuscular free fatty acids [53]. In women with PCOS, peri-muscular adipose tissue in the thigh directly correlated with HOMA-IR and inversely correlated with whole body ISI [35]. Skeletal muscle insulin resistance may develop in part due to increased lipid peroxidation and the by-products thereof [54,55,56,57]. Together, these data point to a role for lipids in the skeletal muscle in insulin sensitivity and resistance and likely reflect altered fuel handling when lipids accumulate in the muscle. 

### 3.4. Hyperinsulinemia and Insulin Resistance in PCOS

In our study, the increase in lipids in the mixed composition gastrocnemius and tibialis anterior muscles, coupled with higher fasting insulin levels and diminished phosphorylation of total IRS1 and p70S6K, appear to reflect increased skeletal muscle insulin resistance. Hyperinsulinemia and insulin resistance are common in adolescent and adult women with PCOS [23,24,25,26]. These findings have been reproduced in animal models for the disorder, both in PNA models and models using postnatal induction of a PCOS phenotype [11,30,58,59,60,61]. The model used in this study has previously been reported to exhibit metabolic dysfunction at 8.5 weeks of age, including greater parametrial and subcutaneous adipose as a percentage of body weight, elevated serum triglycerides and cholesterol, and elevated fasting serum insulin with no significant difference in responses to a glucose tolerance test (GTT) [32]. In the present study, we focused on the skeletal muscle changes in this model and confirmed fasting hyperinsulinemia at 16 weeks of age prior to investigating expression of insulin signaling proteins in this fasting, hyperinsulinemic state. Hyperinsulinemia is reported to precede insulin resistance and weight gain in at least one animal model for PCOS [62] but may also result from tissue insulin resistance and compensation to maintain glucose homeostasis [63,64]. Elevated androgens in females may promote insulin resistance, highlighting the reciprocal relationship between insulin and androgens [36,63,65,66,67,68,69]. Insulin resistance might not be conserved in cells derived from women with PCOS [70] and may be adaptive and dependent upon the hormone milieu of the cells and tissues [71].

There are tissue-specific differences in PCOS-related insulin resistance, with skeletal muscle and myotubes derived from women with PCOS demonstrating insulin resistance and decreased insulin responsiveness, as well as adipocytes with impaired insulin sensitivity but normal responsiveness [72,73]. Both acquired and intrinsic defects in skeletal muscle from women with PCOS are reported to contribute to insulin resistance in this tissue [71,74,75]. Impaired phosphorylation of Akt and AS160 in response to insulin [76] and impaired activation and dephosphorylation of glycogen synthase [77] have been reported in skeletal muscle from women with PCOS, underscoring the impact on the metabolic arms of the insulin signaling pathway. Activity of the ERK1/2 component of the mitogenic arm of the insulin signaling pathway in skeletal muscle is reported to be upregulated in women with PCOS, resulting in phosphorylation predominantly at Ser312, which may then result in reduced activation of the metabolic portions of the pathway and insulin resistance [78]. In our study, we examined the expression of total and Ser^636/9^-phosphorylated IRS1 and found an apparent decrease in the ratio of the phosphorylated form relative to total IRS1 in gastrocnemius and tibialis anterior muscles; together with increased lipid content, these findings are consistent with insulin resistance in these muscles. Phosphorylation at this site is reported to play a role in activation of the mTOR pathway and upregulating protein synthesis [79]. In the soleus muscle, phosphorylation of IRS1 at Ser^636/9^ was increased, which may reflect insulin sensitivity and greater activation of the pathway in the presence of elevated fasting insulin levels. We also examined total and Thr389-phosphorylated p70S6K and found a decrease in the level of phosphorylated p70S6K. Phosphorylation at this site is reported to best correlate with activity of the kinase, which phosphorylates the S6 protein of the 40S ribosome and is required for cell growth and G1 phase progression [80,81]. 

### 3.5. Summary and Future Perspectives

Together, our findings suggest that this PNA model for PCOS results in hyperinsulinemia and skeletal muscle insulin resistance that is due in part to reduced Ser^636/9^ IRS1 and T^389^ p70S6K phosphorylation in some of the mixed composition hindlimb muscles. This may result in a phenotypic shift toward more fast oxidative fibers and, in conjunction with greater muscle lipid content, suggests altered fuel usage. Future perspectives include examination of muscle force transduction and fatigability in this model, and further examining the mechanisms underlying the fiber shift and fuel usage under different physiological and pathophysiological conditions, in order to gain a more comprehensive understanding of the changes in skeletal muscle of PNA rats. Studies on other markers of metabolic dysfunction are also warranted to gain further insights into the pathophysiologic mechanisms in this rodent model for PCOS. In addition, the effects of interventions aimed at improving insulin sensitivity in women with PCOS (e.g., regular aerobic exercise and insulin-sensitizing drugs) on skeletal muscle structure and function are warranted. Such studies would provide mechanistic insight into the effectiveness of these therapies and may guide the development of additional therapeutic options. 

## 4. Materials and Methods 

### 4.1. Materials

All chemicals and reagents were purchased from Sigma Aldrich (St. Louis, MO, USA) unless otherwise noted.

### 4.2. Animals

All procedures involving rats were approved by the Lake Erie College of Osteopathic Medicine Institutional Animal Care and Use Committee (#17-05, 1 September 2017). Sprague–Dawley rats used for breeding were purchased from Hilltop Lab Animals, Inc. (Scottdale, PA, USA). All rats were given ad libitum access to tap water and standard rat chow (Hilltop Lab Animals, Inc.). Rats were housed in a temperature-controlled animal facility (23–25 °C) with a 12 h light/dark cycle. 

### 4.3. Prenatal Androgenization of Rats

Each breeding pair was housed together in a mating cage until the presence of a vaginal plug confirmed pregnancy (gestational day 1, GD1). The male and female breeders were then housed separately. On the mornings of gestational days 16–19, the pregnant dams were subcutaneously injected with 5 mg testosterone dissolved in 500 µL of a 4:1 sesame oil-benzyl benzoate mixture (T rats) or 500 µL of the vehicle alone (SO rats) [31,32,33]. Pups were weaned and separated by sex at 3 weeks of age and then housed 2–3 rats per cage. 

### 4.4. Weekly Body Weight Measurements

Beginning at 3 weeks of age and continuing through 16 weeks of age, female rats were weighed. The weights were recorded on the same day each week (e.g., 3 weeks and 0 days of age).

### 4.5. Rota-Rod Treadmill Test

At 15 weeks of age, rats were introduced to the rota-rod treadmill (Med Associates Inc., St. Albans, VT, USA) and allowed to walk on the wheel, which gradually accelerated from 4.0 rpm to a maximum speed of 40 rpm, for 300 s. The next 3 consecutive days were timed trials. The rota-rod stopped when the rat fell off the wheel and broke the photo beam at which point the time spent on the apparatus was recorded, or after 1000 s (~16.5 min, the maximum time allowed on the rota-rod). Durations of less than 60 s were counted as failed trials; for failed trials, rats were given a maximum of 3 trial attempts each day. Timed trials were recorded for each rat for 3 consecutive days and averaged for each rat.

### 4.6. Blood Collection and Fasting Blood Glucose

At 16 weeks of age, rats were fasted for 10 h overnight. In the morning, rats were anesthetized with an intraperitoneal injection of 80 mg/kg thiobutabarbital in normal saline. A tail vein blood sample was collected using a 25G butterfly needle and syringe. A drop of blood was used to determine blood glucose levels using a standard glucometer (OneTouch Ultra2; LifeScan, Inc., Wayne, PA, USA). Blood samples coagulated for 10–20 min on ice then were separated by centrifugation at 3000 rpm for 10 min. The serum was then transferred to a clean microfuge tube and stored at −80 °C until thawed on ice for ELISA analysis.

### 4.7. Insulin ELISA

Serum samples from 16-week-old rats (15 SO rats and 9 T rats) were used for analysis of serum insulin by ELISA (Cayman Chemicals, Ann Arbor, MI, USA; cat. 589501). Undiluted samples were analyzed in duplicate within a single assay. The manufacturer’s protocol was used to conduct the analysis and analyze the data. The intra-assay coefficient of variation was less than 10%. 

### 4.8. Tissue Collection

At 16 weeks of age, following the tail vein blood draw under anesthesia, rats were euthanized by cervical dislocation. The tibialis anterior (TA), extensor digitorum longus (EDL), soleus (SOL), and gastrocnemius (GN) muscles were dissected from each animal. Whole muscles were weighed, snap-frozen in liquid nitrogen, then stored at −80 °C. 

### 4.9. Histological Examination of Skeletal Muscles

Frozen skeletal muscle samples from both groups were serially cryosectioned in cross-section at 10 µm thickness, mounted on a glass slide, then fixed in 4% paraformaldehyde in phosphate-buffered saline (PBS). Sections were stained with hematoxylin and eosin prior to mounting a glass coverslip. Tissue sections were observed and imaged using a light microscope at 200× (Olympus CKX41, Olympus, Center Valley, PA, USA) by an investigator blinded to the treatment group.

### 4.10. Measurement of Skeletal Muscle Fiber Cross-Sectional Area

ImageJ software (Version 1.52a; NIH, Bethesda, MD, USA) was used to determine average muscle fiber cross-sectional area from 3 representative fields of tissue for each animal. Tissues from eight animals per group were used for measurement by an investigator blinded to the treatment group.

### 4.11. Immunolabeling and Microscopy

Skeletal muscle samples from both groups were cryosectioned, immunolabelled, and imaged at the same time and using the same conditions and microscope settings. Samples of frozen muscles were cryosectioned in 20 µm slices and mounted on a glass slide. Tissue sections were fixed in 4% paraformaldehyde in PBS, blocked in 3% bovine serum albumin (BSA) with 0.1% Tween-20 in PBS for 1 h at room temperature, then incubated with primary antibodies in 3% BSA in PBS overnight at 4 °C. Primary antibodies used at a 5 µg/mL dilution were: mouse anti-ATP synthase alpha (ThermoFisher, cat. no. 459240 [82]), mouse anti-slow skeletal myosin heavy chain (Abcam, cat. no. ab11083 [83]), and rabbit anti-fast skeletal myosin heavy chain (Abcam, cat. no. ab228727). Tissue sections subsequently washed and incubated with fluorophore-conjugated secondary antibodies in 3% BSA in PBS for 45 min in the dark at room temperature. Secondary antibodies used at a 1:200 dilution were: DyLight 488 goat anti-rabbit [84] or horse anti-mouse [85] and DyLight 549 horse anti-mouse [86] or goat anti-rabbit [87] (Vector Laboratories, Burlingame, CA, USA). Mounting media containing DAPI (Southern Biotech, Birmingham, AL, USA) was used to mount coverslips prior to imaging at 200x (Olympus CKX41). Two representative fields from each slide were imaged and quantified by an investigator blinded to treatment group. Immunofluorescence was quantified from an 8-bit image using ImageJ software (Version 1.52a).

### 4.12. Skeletal Muscle Fiber Typing

Muscle sections were immunolabeled with antibodies to the fast and slow isoforms of myosin heavy chain indicated above. Muscle fibers in 3 distinct fields for each muscle were imaged and categorized as positive for fast myosin heavy chain (fMHC+), slow myosin heavy chain (sMHC+), or both (f,sMHC+) by an investigator blinded to the treatment group.

### 4.13. Oil Red O Staining of Lipids

Skeletal muscle samples from both groups were serially cryosectioned in cross-section at 10 µm thickness, mounted on a glass slide, then fixed in 4% paraformaldehyde in phosphate-buffered saline (PBS). Sections were washed 3 times in deionized water after 30 min in paraformaldehyde, then incubated as follows: 60% isopropanol for 5 min, Oil Red O solution for 20 min, washed 5 times in deionized water, hematoxylin for 1 min, washed 5 times again in deionized water, then a coverslip mounted with aqueous mounting medium. Tissue sections were observed and imaged using a light microscope at 200× (Olympus CKX41) by an investigator blinded to the treatment group.

### 4.14. Western Blot Analysis

Samples of muscle tissue were homogenized in ice-cold buffer (10 mM Tris pH 7.4, 150 mM NaCl, 5 mM EDTA, 1% Triton X-100, 1% Sodium Deoxycholate, 0.1% SDS, with Halt^TM^ protease and phosphatase inhibitor (ThermoFisher, Waltham, MA, USA)), and a 21G needle was used to shear genomic DNA prior to centrifugation at 13,000 rpm for 10 min. Total protein concentration determined by BCA assay (ThermoFisher). Tissue homogenates (30 µg) were separated by SDS-PAGE and proteins transferred to nitrocellulose prior to blocking with 4% bovine serum albumin (BSA) in Tris-buffered saline (TBS). Blots were incubated with primary antibodies in 4% BSA in TBS with 0.1% Tween-20 overnight at 4 °C. Primary antibodies used were: mouse anti-ATP synthase alpha (ThermoFisher, cat. no. 459240 [82], 2 µg/mL) and mouse anti-GAPDH (ABclonal, cat. no. AC002 [88], 1:5000); and from Cell Signaling Technology, rabbit anti-phospho-IRS1 S^636/9^ (cat. no. 2388 [89], 1:500), rabbit anti-IRS1 (cat. no. 2382 [90], 1:1000), rabbit anti-IR-beta (cat. no. 3025 [91], 1:1000), rabbit anti-phospho-Akt S^473^ (cat. no. 9271 [92], 1:500), rabbit anti-Akt (cat. no. 9272 [93], 1:1000), rabbit anti-phospho-p70S6K T^389^ (cat. no. 9205 [94], 1:1000), and rabbit anti-p70S6K (cat. no. 9202 [95], 1:1000). IRDye^®^ secondary antibodies, anti-mouse-680RD [96], and anti-rabbit-800CW [97], were diluted 1:20,000 in Intercept TBS buffer with 0.2% Tween-20 (LI-COR, Lincoln, NE, USA). Blots were imaged with a LI-COR Odyssey Fc imager (LI-COR, Lincoln, NE, USA), then quantified and analyzed using Image Studio Lite software (Version 5.2; LI-COR, Lincoln, NE, USA).

### 4.15. Statistical Analysis

GraphPad Prism 7 software (Version 7; San Diego, CA, USA) was used for statistical analysis. All data were tested for normality (D’Agostino Pearson test for normality) prior to statistical analysis with the appropriate parametric or non-parametric test. One-way ANOVA with Sidak’s multiple comparisons test was used to analyze body weight. Unpaired 2-tailed t-tests were used to analyze rota-rod time trials, weights of EDL and soleus muscles, muscle fiber cross-sectional area, muscle fiber composition for the gastrocnemius and TA muscles, and Western blot densitometry. Mann–Whitney U tests were used to analyze weights of TA muscles, muscle fiber composition for the soleus, ATP synthase immunofluorescence intensity, fasting glucose and insulin, and Western blot densitometry for F1-ATP synthase (gastrocnemius and soleus muscles) and IRS1 (soleus muscle). Values reported are the means with standard deviation (for data analyzed with a parametric test) or medians with quartiles (for data analyzed with a non-parametric test). *p* < 0.05 was considered significant. *p* values between 0.05 and 0.10 were considered trending. 

## 5. Conclusions

PNA female rats exhibited larger muscle mass and fiber CSA, as well as a shift towards more fibers exhibiting a fast oxidative glycolytic phenotype in the GN muscle. Elevated fasting serum insulin levels may be an early indicator of skeletal muscle insulin resistance in this model and corresponded with increased IRS1 protein levels and a reduced ratio of Ser^636/9^ phosphorylated IRS1 to total IRS1 in the GN and TA muscles, as well as decreased Thr^389^ phosphorylated p70S6K in GN. More lipid content in the PNA GN and TA is consistent with insulin resistance in these muscles. The shift towards a fast oxidative glycolytic fiber phenotype may be an adaptive mechanism to impaired insulin sensitivity or altered fuel availability.

## Abbreviations

CSA	cross-sectional area
EDL	extensor digitorum longus
fMHC	fast isoform of myosin heavy chain
fMHC+	positive for fast isoform of myosin heavy chain
GN	gastrocnemius
HOMA-IR	homeostatic model assessment of insulin resistance
MHC	myosin heavy chain
PCOS	polycystic ovary syndrome
PNA	prenatally androgenized
sMHC	slow isoform of myosin heavy chain
sMHC+	positive for slow isoform of myosin heavy chain
SO	sesame oil
SOL	soleus
T	testosterone
TA	tibialis anterior
